# Risk Factors and Preventive Measures for Lung Cancer in the European Union

**DOI:** 10.3390/epidemiologia5030037

**Published:** 2024-08-27

**Authors:** Katharina Kehrle, Michael Hetjens, Svetlana Hetjens

**Affiliations:** 1 Department of Medical Statistics and Biomathematics, Medical Faculty Mannheim, University of Heidelberg, Theodor-Kutzer-Ufer 1-3, D-68167 Mannheim, Germany; katharinakehrle@web.de; 2 Department of Biomedical Informatics, University of Heidelberg, Theodor-Kutzer-Ufer 1-3, D-68167 Mannheim, Germany; michael.hetjens@medma.uni-heidelberg.de

**Keywords:** prevention, lung cancer, mortality

## Abstract

Background: Lung cancer is worldwide one of the most common types of cancer with still very high mortality rates. The aim of this study was to identify and demonstrate correlations between lung cancer mortality rates and potential influencing factors in EU countries. Methods: This retrospective study investigated the connections between the mortality rates in the EU countries (n = 28) and potential influencing factors. The significant factors from the correlation analysis were identified using a stepwise multiple regression analysis. Results: The most important factors for both genders are the incidence of lung cancer, the price of tobacco, and the number of doctors per 100,000 inhabitants. Conclusion: Lung cancer is a significant global health challenge. The study identified potential strategies for reducing the mortality rate from lung cancer. These strategies include an increase in the number of physicians, enhanced accessibility to cutting-edge antineoplastic medications, and state-funded coverage of the associated costs. It would be beneficial for politicians to consider implementing LDCT screening for the early detection of the disease. The implementation of uniform healthcare system optimization across the EU, combined with improvements in socio-economic conditions, has the potential to mitigate the risk of developing lung cancer.

## 1. Introduction

Lung cancer is the most commonly diagnosed cancer worldwide. In 2020, approximately 2.2 million new cases of lung cancer were diagnosed globally [1]. Lung cancer is responsible for about 1.8 million deaths per year. Non-small cell lung cancer (NSCLC) is more common and grows slowly, while small cell lung cancer (SCLC) is less common but often grows quickly [2].

The most important risk factor for lung cancer is smoking. About 85% of lung cancer cases are attributed to smoking [2]. Passive smoking is also recognized as a risk factor [3]. Air pollution, particularly fine particulate matter (PM2.5), is also a significant risk factor. Exposure to certain chemicals and substances (e.g., asbestos, radon) increases the risk of lung cancer. Occupational exposure to carcinogenic substances accounts for approximately 9–15% of all lung cancer cases [4]. Numerous studies also confirm a link between lung cancer and viruses, such as human papillomaviruses [5,6].

The most important primary prevention measure against lung cancer is tobacco control and smoking prevention. Lung cancer screening with low-dose computed tomography (LDCT) can reduce mortality by detecting cancer at an earlier, more treatable stage [2]. Approximately 90% of patients with lung carcinoma initially exhibit non-specific symptoms. These primarily include cough, weight loss, dyspnea, chest pain, hemoptysis, bone pain, clubbing, fever, weakness, superior vena cava obstruction, dysphagia, wheezing, and stridor [7]. Often, there are delays of several weeks or even months before the diagnosis is made.

The following examinations are included in the basic diagnostics [8]:

- Medical history and clinical examination;
- Laboratory tests (blood count including liver and kidney parameters, coagulation values, and electrolytes);
- Chest X-ray in two planes (initial imaging procedure);
- Spiral CT of the thorax (contrast agent in the portal venous phase);
- Bronchoscopy;
- Abdominal ultrasound.

The treatment of lung cancer depends on the stage and type of cancer and may include surgery, radiation therapy, chemotherapy, targeted therapy, and immunotherapy [2]. If NSCLC is detected at an early stage and the tumor is completely resected, the 5-year survival rate significantly improves to 72% in stage IA, 59.8% in stage IB, 45% in stage IIIA, and 38.7% in stage IIIB [9]. For 60–70% of patients, SCLC is diagnosed at advanced stage IV. Only 5% of these patients are alive two years after diagnosis [10].

The aim of this study was to identify and demonstrate correlations by analyzing mortality data and various potentially influential factors in the countries of the European Union (EU). The presented relationships could thus facilitate a more comprehensive understanding of etiological risk factors and potential intervention points for the prevention of lung cancer.

## 2. Material and Methods

For this study, the mortality data from the WHO “mortality” database were selected for the following ICD-10 code C34 for lung cancer. Due to the different coding habits of the EU countries, the ICD codes C34.0 to C34.9 have been summarized. In order to obtain a comprehensive picture of the factors influencing the etiology within the EU countries, this study not only analyzed some classic medical risk factors for lung cancer, but also the circumstances and living conditions in the respective countries. Potential influencing factors on lung cancer mortality rates were selected from the WHO “European Health for all (HFA) database”:

Factors related to the healthcare system and disease-related factors are as follows:

- Hospitals per 100,000 inhabitants;
- Doctors per 100,000 inhabitants;
- Primary healthcare units per 100,000 inhabitants;
- Healthcare expenditure as % of GDP;
- Healthcare expenditure per capita, US dollars;
- Public sector healthcare expenditure as % of total healthcare expenditure;
- Pharmaceutical expenditure per capita, US dollars;
- Number of new cases of lung cancer;
- Incidence of bronchial carcinoma per 100,000, men;
- Incidence of bronchial carcinoma per 100,000, women.

Social and economic factors are as follows:

- Average population density per km^2^;
- Unemployment rate in %;
- Gross national income per capita, US dollars;
- Gini coefficient (measure of income inequality or wealth inequality).

Individual factors are as follows:

- Regular daily smokers from age 15 and older in %;
- Age-standardized prevalence of overweight, from age 18 and older in %;
- Alcohol consumption in liters per capita, from age 15 and older;
- Age-standardized prevalence of smokers in % from age 15 and older;
- Annual per capita cigarette consumption;
- Private household expenditure on health as % of total healthcare expenditure.

Environmental factors are as follows:

- Average annual concentration of sulfur dioxide in µg/m^3^ in the capital;
- Average annual concentration of fine particulate matter < 10 µg in µg/m^3^ in the capital;
- Average annual concentration of nitrogen dioxide in µg/m^3^ in the capital;
- Average annual concentration of ozone in µg/m^3^ in the capital.

Even though the etiology of bronchial carcinoma is multifactorial, smoking is the risk factor with the greatest significance. Therefore, the parameter Tobacco Control Scale (TCS) from the “Association of European Cancer Leagues” was analyzed. In a point system with a maximum of 100 points, European countries were evaluated based on their activities in tobacco control and smoking prevention. The points were awarded in six areas: cigarette price, smoke-free environment, spending on campaigns, advertising bans, warning labels, and smoking cessation [11]. The tobacco price was selected from the “Statistics Explained” database. In order to be able to estimate the radiotherapy care in the countries, the data were extracted from the DIRAC (Directory of Radiotherapy Centers) database.

The countries’ data were analyzed for the period 2010 to 2014 (as no patient data were used, no ethical vote was required). The research was conducted in 2017, at which time the data up to 2014 were available in the WHO databases. The 5-year period was chosen to determine trends. Over a longer period, there are many changes in therapies and policies. These changes can only be insufficiently considered in the models. The 5-year period was chosen to adapt to this problem. Direct age standardization of the mortality data of the EU countries (n = 28) was carried out to exclude the possibility that the mortality differences between these countries were due to different age structures. Germany’s population was used as the standard population. The following classes (k) were used: k1 = 1–14, k2 = 15–24, k3 = 25–49, k4 = 50–69, and k4 = over 70. Correlation analysis was used to determine the relationships between the selected quantitative factors and mortality rates. A multiple stepwise regression analysis was then performed on the significant factors in the correlation analysis to evaluate multiple influencing factors. The standard SAS criteria were used for this: The significance level for staying in the model was specified as 0.15 and the significance level for entry into the model was specified as 0.5. The presence of collinearity in the regression was verified through the utilization of the SAS option COLLIN. In addition, this analysis provides a test variable (F) per parameter, according to which the *p*-value is calculated. The larger this value, the more important is the parameter in the model. And, therefore, the most important factor with the most influence on the dependent parameter (mortality rate). Multiple imputation for missing data was used for this analysis. This was conducted using the SAS procedure PROC MI and FCS statement (FSC uses the regression method). The *p*-value of <0.05 was considered significant as the statistical significance level. Analysis was performed using SAS version 9.3 (SAS Institute, Cary, NC) software.

## 3. Results

### 3.1. Factors

The average lung cancer mortality rate for women in the EU in the years 2010 to 2014 was 3.37 people per 10,000, while the average mortality rate for men was 8.87 people per 10,000. In this study, correlations between the mortality rates in the EU countries (n = 28) and potential influencing factors were investigated. The significant factors were then further analyzed using a stepwise regression analysis. The results of the collinearity analysis indicated the absence of any conspicuous values.

Based on the F-value for women, besides the incidence of lung cancer, the number of doctors per 100,000 inhabitants is the most important factor, followed by tobacco price. (Table 1).

For men, the most important factors are the incidence of lung cancer, the price of tobacco, and the number of doctors (Table 2).

### 3.2. Preventive Measures

To develop a possible strategy for the prevention of lung cancer, research was carried out on the three possible levels of prevention (primary, secondary, and tertiary).

The most important primary prevention measure against lung cancer is undoubtedly tobacco control and smoking prevention. 

The most important strategies for secondary prevention of lung cancer are early detection and screening. People with an increased risk of lung cancer can be identified by the PLCOm2012 model [12]. To assess the individual risk of developing lung cancer, this model considers the following 12 predictors:

- Age;
- Socioeconomic status;
- Family history of lung cancer;
- Body Mass Index (BMI);
- COPD;
- Smoking status;
- Duration of smoking;
- Smoking intensity;
- Time since quitting smoking;
- Other cancer diagnoses;
- Ethnic group;
- Chest X-ray in the past 3 years.

Through computed tomography screening in individuals with increased risk for lung cancer, the tumor can be detected at an early stage [13,14]. 

The most important strategies for tertiary prevention of lung cancer are treatment and therapy. Current drug therapies for lung cancer can be divided into chemotherapeutic agents and new antibody-based cancer medications, also known as EGFR tyrosine kinase inhibitors. These treatments aim less for cure and more for prolonging survival and/or improving quality of life.

The European Society for Medical Oncology (ESMO) published a study in 2016 on the availability, accessibility, and cost of antineoplastic drugs in Europe, with the following results. Most relevant chemotherapeutic agents are widely available across Europe and associated with low costs for patients [15]. Access to the new antibody-based cancer medications, however, varies significantly across Europe. The medications examined in the ESMO study include Erlotinib, Gefitinib, Afatinib, and Crizotinib. While these drugs are largely available and subsidized in Western Europe, they are either unavailable, or accessible only at high costs, to individual patients in Eastern Europe. This disparity is largely attributed to the continuously rising costs of cancer medications. For instance, the average price of new cancer medications has more than doubled from USD 4500 to over USD 10,000 over the past decade [15].

## 4. Discussion

Lung cancer leads to around 1.82 million deaths annually worldwide, making it the leading cause of cancer deaths globally. In this study, potential influencing factors on lung cancer were selected from the HFA WHO database and correlated with the mortality rate of lung cancer from the WHO mortality database. Data from 2010 to 2014 from the 28 EU countries were analyzed. In particular, the Tobacco Control Scale parameter from the Association of European Cancer Leagues was analyzed. The period of five years is limited; therefore, only trends can be described. Furthermore, the following limitations are evident: Missing values in the regression analysis were addressed through the use of multiple imputation, as this was the sole method by which all countries and factors could be analyzed. However, multiple imputations represent only estimated values. The reliability of regression analyses is impaired by possible distortions, confounding factors, and unmeasured variables. These could cause distortions and even result in factors that are not significant being identified as significant and vice versa. Despite the absence of any conspicuous values in the collinearity analysis, it is possible that the factors included in the model may be interdependent, particularly those pertaining to smoking.

The most important factors for both genders are the incidence of lung cancer, the price of tobacco, and the number of doctors per 100,000 inhabitants. Tobacco consumption is the largest avoidable behavioral risk factor for lung cancer. The enormous influence of tobacco prices on young people remains undisputed, as they are up to three times more sensitive to a price increase than adult smokers [16]. Since 52% of all smokers in the EU start smoking before the age of 18, it becomes increasingly difficult to quit as they get older. Raising tobacco taxes can literally tackle the problem at its root and significantly reduce the incidence of smokers [17]. Therefore, the countries should make greater use of the tool of tobacco price increases. It is also important to provide comprehensive and free counseling and treatment for smoking cessation. In addition to the challenge of reducing the number of smokers in the population, it is also important to protect non-smokers from passive smoke through appropriate non-smoking protection laws in public places and workplaces.

The healthcare systems of different countries in the European Union vary significantly in terms of organization, structure, and financing. There is a divergence in the content, scope, and methodological rigor of lung cancer care across Europe [18]. Comparing these different health care systems is therefore very complex. Various organizations, including the WHO, the Organization for Economic Cooperation and Development (OECD), and the European Commission and the Health Consumer Powerhouse, regularly publish assessments of the health systems of European countries. Since 2005, the latter has attempted to evaluate and compare healthcare systems on an annual basis with the so-called Health Consumer Index. The assessment is based on a maximum of 1000 points awarded for 46 factors in the following 6 areas: patients’ rights, accessibility (waiting times for treatment), outcomes, range and accessibility of services, prevention, and medicines. The results of the current Health Consumer Index show parallels with the standardized mortality rates for lung cancer [19]. Healthcare systems in different countries differ significantly in terms of objectives and priorities. Harmonization is therefore a difficult and complex process. This can be clearly seen in the adaptations to healthcare systems as a result of the global pandemic (COVID-19). Many countries focused on maintaining basic healthcare services and protecting vulnerable population groups. Others focused on improving management capacity, preparing staff, using digital health technologies, and strengthening primary healthcare systems [20]. The relationship between physician density and treatment success is not well studied. A study from the year 2000 concluded that a 10% increase in the number of physicians reduces the premature mortality rate of women by 4% and of men by 3%. This study examined, among other factors, mortality rates and key aspects of medical care from 1970 to 1995 in 21 member states of the Organization for Economic Co-operation and Development (OECD) [21]. Another study showed that an increase in the density of general practitioners by 10 physicians per 100,000 inhabitants could reduce cancer mortality by 1 percentage point and mortality from respiratory diseases by 1.4 percentage points [22]. 

In the area of social and economic factors, the relationship between the unemployment rate and mortality rate has been examined, among other aspects. The results of statistical analyses, particularly for men, indicate that a rising unemployment rate is associated with an increasing mortality rate. The correlation between socioeconomic status and lung cancer mortality has been previously demonstrated in other studies. [23,24]. Additionally, an increase in unemployment has been observed concurrently with an increase in other types of cancer [25]. This further underlines the importance of access to healthcare.

Regarding individual factors, obesity and alcohol consumption correlate with the mortality rate only in males. A 2005 study confirms the differing results for the factor of alcohol consumption between men and women. While non-smoking women with frequent alcohol consumption did not show an increased risk of lung cancer, an increased risk was observed in men [26]. Obesity is a relevant risk factor for numerous types of cancer [27]. However, bronchial carcinoma appears to be an exception. A high BMI is associated with a reduced risk of lung cancer and an improved survival rate [28]. A differentiated examination of non-smokers and lung cancer patients between the sexes seems useful for future studies.

The prevention measures presented resulted in the following findings: The most important prevention measure in the fight against lung cancer is tobacco consumption control. The prediction model PLCOm2012 could identify people who have a high risk of lung cancer. Through computed tomography screening, individuals at increased risk for lung cancer can be detected at an early stage. Current drug therapies for lung cancer could be divided into chemotherapeutic and new antibody-based cancer drugs. In 2018, Hirsch et al. published the first long-term study on treatment with Gefitinib, with an average treatment duration of 11.1 years. The 10-year survival rate of patients initially treated with Gefitinib was 86%, and the 15-year survival rate was 59% [29]. Therapy with tyrosine kinase inhibitors can extend patient survival and this can reduce the mortality rate. In particular, access to the new antibody-based cancer drugs greatly varies across Europe and should be improved.

## 5. Conclusions

The results of this study provide EU countries with new strategies to reduce lung cancer mortality. Key contributing factors include increasing the number of physicians, improving the accessibility of new therapeutic agents, and providing government subsidies for the associated costs. In addition to established tobacco prevention measures, it is recommended to introduce LDCT screening for early detection of patients. The implementation of uniform healthcare system optimization across the EU, combined with improvements in socio-economic conditions, such as access to education, healthcare, and healthier living conditions, has the potential to mitigate the risk of developing lung cancer.

## Figures and Tables

**Table 1:** Multiple regression for lung cancer in women, R^2^ = 0.9573.

Parameter	*p*-Value	F-Value
Incidence rate per 100,000 women	<0.0001	1552.63
Doctors per 100,000	<0.0001	65.58
Tobacco price	<0.0001	57.17
Tobacco control scale	<0.0001	55.55
Daily smokers %	<0.0001	52.67
Health expenditure % of GDP	<0.0001	48,3
Total health expenditure GDP per capita	<0.0001	31.49
Prevalence of smokers %	<0.0001	28.37
Unemployment rate %	<0.0001	19.47
GINI coefficient	0.0038	8.7

**Table 2:** Multiple regression for lung cancer in men, R^2^ = 0.9513.

Parameter	*p*-Value	F-Value
Incidence rate per 100,000 men	<0.0001	981.38
Tobacco price	<0.0001	129.29
Doctors per 100,000	<0.0001	72.52
Health expenditure % of GDP	<0.0001	64.58
Prevalence of overweight	<0.0001	43.38
Unemployment rate %	<0.0001	40.8
Total health expenditure GDP per capita	<0.0001	16.65
Gross national income per capita	0.0003	14.02
Hospitals per 100,000	0.0124	6.44
Prevalence of smokers %	0.0131	6.33
Prevalence of smokers %, men	0.0302	4.81
Radiation therapy capacity	0.0390	4.35

## Data Availability

The data presented in this study are available upon request from the corresponding author.
